# Allulose for the attenuation of postprandial blood glucose levels in healthy humans: A systematic review and meta-analysis

**DOI:** 10.1371/journal.pone.0281150

**Published:** 2023-04-06

**Authors:** Yuma Tani, Masaaki Tokuda, Naoki Nishimoto, Hideto Yokoi, Ken Izumori

**Affiliations:** 1 Matsutani Chemical Industry Co. Ltd., Itami-city, Japan; 2 Kagawa University, Takamatsu-city, Japan; 3 Clinical Research and Medical Innovation Center, Hokkaido University Hospital, Sapporo-city, Japan; 4 Department of Medical Informatics, Kagawa University Hospital, Kita-gun, Japan; 5 Faculty of Agriculture, Kagawa University, Kita-gun, Japan; Bangor University, UNITED KINGDOM

## Abstract

D-Allulose is a rare sugar that exists in nature. It is a food ingredient with nearly zero calories (<0.4 kcal/g) and has many physiological functionalities such as attenuation of postprandial blood glucose levels, attenuation of postprandial fat mass accumulation, and anti-aging property. This study focused on the postprandial blood glucose changes in healthy humans by a systematic review and meta-analysis. They were chosen because of its importance to a prevention from diabetes. The study objective was to examine acute blood glucose concentrations of healthy humans after the meal with and without allulose. The study collected all D-allulose related studies from various databases. A forest plot of the comparison between an allulose intake group and the control group showed both 5g and 10g intake groups have the significantly smaller area under the curve of postprandial blood glucose levels. It means that D-Allulose attenuates postprandial blood glucose concentrations in healthy humans. As the result, D-Allulose is a valuable blood glucose management tool for healthy humans and diabetes patients. Allulose Diet enables reduction of sucrose intake through Sugar Reformulation in the future diet.

## 1. Introduction

According to the World Health Organization (WHO), noncommunicable diseases (NCDs) kill 41 million people each year and accounts for about 70% of all deaths globally. More than three quarters of global NCD deaths occur within low—middle income countries. They have indicated that there are four key metabolic changes that increase the risk of NCDs; they are, "raised blood pressure", "overweight/obesity", "hyperglycemia (high blood glucose levels)" and "hyperlipidemia (high levels of fat in the blood)" [1].

WHO issued a guideline to reduce the risk of NCDs. The guideline recommends “a reduced intake of free sugars throughout the lifecourse” [2]. However, sugars are the major source of energy which provide preferable sweet taste to humans in all kinds of foods. In other words, "Sugar Reduction" is something that we have not accomplished so far. It is the one of the reasons for such prevalence of the NCDs worldwide.

In order to reduce sugar intake, food industries have been trying to find alternative materials to replace sugar’s sweet taste with a low-calorie material under the concept of "Sugar Substitution".

There are a series of discoveries and developments within the sweetener industries. The quality and availability of sugars like sucrose, glucose, and fructose has improved with mass production. Saccharin, aspartame, acesulfame potassium, sucralose, monk fruit and stevia are high potency sweeteners that have been utilized. Sorbitol, maltitol, erythritol, and xylitol are sugar alcohols that are also available options that provide sweetness. All those different materials have been tested and developed based on sucrose as a benchmark material, comparing taste, appearance, smell, and other attributes of sucrose. There is no perfect fit for the Sugar Substitution.

The theory is that all sugars have a caloric value of about 4 kcal/g and provide the same amount of energy and other criteria as sucrose just like glucose and fructose. This assumption doesn’t apply to recently discovered monosaccharides. Tagatose is known as a low calorie monosaccharide of 1.5kcal/g [3].

Dr. Izumori and his research group have found a new way to manufacture a monosaccharide called D-allulose (D-ribo-2-hexulose; CAS registration number: 551-68-8; molecular formula: C6H12O6; molecular weight: 180.156) here in after known as “allulose” by a newly found enzyme in the early 90’s [4]. Allulose exists rarely in nature as a part of some plants like *Itea* [5]. Its low abundance in nature made its discovery of taste and other properties difficult. Because of that, allulose is categorized within the category of "Rare Sugars" today [6]. It is rare, yet common in some ways as most people eat small amounts of allulose in various food products like ketchup, caramels, and raisins [7]. Accordingly, there is a history of allulose consumption.

Allulose is a C-3 epimer of D-fructose, it is also known as D-psicose. Its appearance as white crystals with sweet taste are similar to sucrose. It is a unique food ingredient, and has an extremely low calorie count as 0–0.39kcal/g calculated from both animal and clinical studies [8, 9] while typical sugars are known to have a caloric value of about 4 kcal/g. Many functionalities, other than a low-calorie value, were discovered [10–12] such as postprandial fat oxidation [13], fat oxidation during exercise [14], anti-obesity/less body fat accumulation [15–19], inhibits the expression of MCP-1 [20], attenuation of glycemic response [21], and even further as anti-aging property [22] other than it being low calorie were discovered to help understand its value. There is a systematic review for health effects [23] as well. Several rat studies indicate that majority of allulose will be excreted in urine and feces [24–26]. Iida et al. found that majority of allulose was excreted in urine within 48 hours and feces from its intake with human patients [9]. It means that there are two routes for administered allulose to be excreted from the body. One route is that allulose is absorbed in the small intestine and enters into the blood stream [9]. Allulose will not raise nor lower the blood glucose levels with a single intake, even though it enters into the blood [27]. About 80% of the allulose is excreted in urine [9]. The remaining allulose passes through the small intestine and reaches the large intestine where negligible fermentation happens and is excreted in feces [9].

There are multiple mechanisms of allulose found as followings. Allulose has been shown to inhibit α-glucosidase activities which leads to suppression of glycemic responses after carbohydrate ingestion [27]. One study indicates slower absorption of glucose if allulose is also present and vice versa [28]. Allulose has been shown to stimulate glycogen synthesis in the liver [29], and promotes faster restoration of glycogen in the liver and muscle after exercise [30]. Allulose induces Glucagon-like peptide-1 (GLP-1) release from intestinal L-cells, and regulates glucose concentrations after glucose and allulose intake [31–34]. There is a preliminary rat study which indicates allulose prevents progression and development of diabetes [35], protects pancreas [36], and translocate hepatic glucokinase [37]. There is a clinical study with type-2 diabetes [38, 39]. Because of above criteria, allulose and other rare sugars are likened to a dream sugar [40]. If that is the case, we no longer need the Sugar Substitution. What we need is a Sugar Reformulation. It is a mixture of sucrose “sugar” and allulose “sugar”, then it will have a huge impact on the annual sugar consumption in the world which is currently more than 176 million metric tons [41]. In recent year, allulose annual market is about 10,000 metric tons, and it’s increasing every year.

Previous trials indicate potential health benefits to humans in acute glycemic responses. Those trials were done in a small number of patients. The studies can be combined to obtain stronger evidence about their benefits as a Systematic Review. A part of one study is presented by Braunstein et al. under the title "Effect of fructose and its epimers on postprandial carbohydrate metabolism: A systematic review and meta-analysis" [42] to see the result for above. It was a well-designed study and covered other rare sugars like tagatose. Its study design is innovative since it shows effect in the homogenous population health status. However, diabetic patients could have been in an existing treatment plan and may not be helpful to see allulose’s true value or actual strength for healthy humans. According to the WHO report [1], prevention and control of NCDs are important. For the prevention, it is more important to provide a way to control blood glucose levels for healthy people. In addition to above, it doesn’t cover some of the Japanese studies even though the majority of rare sugar studies including allulose stem from Japanese research groups i.e. Matsuo et al. reported to Kagawa University [43]. With the above reasons, we set new inclusion/exclusion criteria and additional database search for the study so that we could investigate whether allulose is effective in healthy humans or not.

The study objective is to check acute blood glucose levels of healthy humans after meal with and without allulose intake and to see if allulose has an effect to postprandial blood glucose by using a systematic review and meta-analysis. We can show whether Allulose is a good management tool for controlling blood glucose levels or not through this study. With this effect, we could use allulose not only for the sweetener, but also a functional sugar for the blood glucose controls to be a key ingredient for the Sugar Reformulation.

## 2. Methods

### 2.1 Methods preparation and registration

The study protocol complies with the Declaration of Helsinki (approval in 1964, revision in 2004), and approved by the ethical committee of Matsutani Chemical Industry Co., Ltd under the No. 200210. Its patient consent was waived by the ethics committee since there is no patient assigned for the study. The methods were prepared using the Cochrane Handbook and Review Manager (RevMan) version 5.4 software (The Nordic Cochrane Centre, The Cochrane Collaboration, 2020, Copenhagen, Denmark) and this paper was reported using the preferred reporting items for systematic reviews and meta-analysis (PRISMA) statement [44–48].

The study protocol was registered through “UMIN Clinical Trials Registry, https://www.umin.ac.jp/ctr/” under the UMIN registration ID of UMIN000039586, and opened on March 1, 2020. EndNote version X8.2 software (Clarivate Analytics, 2016, Philadelphia, United States) was used for the literature searches and reference managements. Summary of findings tables were developed using "GRADEpro, https://gradepro.org/".

### 2.2 Criteria for considering studies for this review

Below were the inclusion criteria of the study.

- The study can be found in full report. *
- The study was conducted within the last 50 years. *^2^
- The study patients are human. *^3^
- The study patients are healthy. *^4^
- The study intervention is allulose. *^5^
- The study patients consume some type of meal to increase blood glucose with the intervention. *^6^
- The study takes blood glucose measurements for at least 2 hours. *^7^
- The study reports Area Under Curve (AUC) of blood glucose levels or it could be obtained from the authors’ group. *^8^

* All studies needed to be of good quality so that they had to be in full report.

*^2^ All studies were conducted within the last 50 years so as to ease studies’ identification work, but cover enough studies for collecting all allulose related studies at same time.

*^3–4^ All study subjects were humans and healthy in order to fulfill the study objective.

*^5^ The study intervention was D-allulose in order to follow study objective.

*^6^ The study is looking for an attenuation effect, so that the control group needed to take some type of meal to increase blood glucose concentrations as well as the intervention group.

*^7^ All studies needed to look for a change in acute blood glucose levels, so that they measured for a length of 2 hours in anyhow i.e. blood glucose levels or plasma glucose levels or otherwise.

*^8^ Their AUC were collected for 2 hours to see common measurement of blood glucose levels, and most studies had data in their report.

Below were the exclusion criteria of the study.

- The study is a review or a case study. *^9^
- Part of the study subjects have a certain medical condition and could not obtain data with only healthy subjects. *^10^
- The part of study intervention is D-allulose, but could not obtain data with only D-allulose intervention. *^11^
- The study doesn’t report AUC of blood glucose levels for 2 hours or we could not obtain its data for 2 hours from authors’ group. *^12^

*^9^ When the study was either a review or a case study, then they could not be included as it was either a duplicate or a single case or doesn’t report any experiment i.e. magazine articles or dictionary pages, which had no value to this study.

*^10^ When a portion of the study’s patients were not healthy, it wasn’t a direct reason to exclude the study. It was carefully reviewed to see if we could obtain data from only the healthy patients to follow the study’s objective.

*^11^ When only part of the study intervention was allulose, then it was difficult to separate the results due to allulose or another intervention. More particularly, there was a product called “Rare Sugar Syrup” or “Rare Sugar Sweet” by Matsutani Chemical Industry Co. Ltd., and those Syrups contain allulose and other rare sugars in combination. They were excluded.

*^12^ Some studies measured blood glucose for more than 2 hours of AUC, and the data was collected for 2 hours to match the data with others. In this case, we contacted the authors’ group for further information, and tried to include the study as much as possible. On the other hand, we excluded them if we failed to receive such data from the authors’ group.

Participants were deemed healthy humans if their change in postprandial blood glucose levels were within a normal range when given a meal. The reason for these criteria was to avoid any influence of medications or treatments that many diabetic or obese patients have.

Intervention was allulose intake. Allulose intake must occur with another energy source intake, which must increase blood glucose concentrations as a meal.

Comparison was made between an allulose intake group and a non-allulose intake group. Both groups must eat either an allulose or a non-allulose with same meal. Other sweeteners could be used for a placebo like aspartame or fructose to fulfill the “non-allulose” control.

Outcomes were based on blood glucose levels, which lead to the study objective and the study question below.

The primary study question was the following: In healthy adults, does 10g or less of allulose, added to a carbohydrate-containing meal, lower postprandial AUC glucose, compared with the same meal without allulose, over the postprandial period in an intervention trial setting?

The secondary study question was the following: In healthy adults, does 5g or less of allulose, added to a carbohydrate-containing meal, lower postprandial AUC glucose, compared with the same meal without allulose, over the postprandial period in an intervention trial setting? In other words, is there any difference between the two different doses? For both study questions, we set 5g and 10g as cut offs. The rationale for those numbers were set from the study referenced above for the attenuation of glycemic responses after carbohydrate ingestion [21]. The study used 10% of allulose from total carbohydrates’ intake as addition. Also, previous meta-analysis study done by Braunstein et al. shows availability of data at 5g and 10g dose levels.

### 2.3 Search methods for identification of studies

We performed a literature search for this study up to a search day of April 7, 2022 from the following databases: MEDLINE through PubMed, CENTRAL, EMBASE, ICHUSHI Web, and CINII.

We included Japanese databases for the search with an historical reason. Allulose’s existence was known for many years, but not so many studies were conducted because of its rare availability in nature. It was subsequently classified as one of the "Rare Sugars" at a later year. After a discovery of enzymatic production methods of allulose by Prof. Izumori from Kagawa University in Japan around the 90’s, many studies were then conducted in Japan. Kagawa and Japan became the center of rare sugar research in the world. For the above reason, it was necessary to locate as many studies as possible written in Japan by Japanese. We search those databases using the Japanese language.

Below shows search formulas for electric searches at each database.

For PubMed, “(allulose) OR psicose”. *^13^

For CENTRAL, “allulose OR psicose”. *^13^

For EMBASE, “allulose OR psicose”. *^13^

For ICHUSHI Web, “(Psicose/TH or allulose/AL) or (Psicose/TH or psicose/AL) or A-RU-ROH-SU/AL or (Psicose/TH or PU-SHI-KOH-SU/AL)” *^13^ *^14^

For CINII, “allulose OR psicose OR A-RU-ROH-SU OR PU-SHI-KOH-SU” *^13^ *^14^

*^13^All search formula is exactly typed within the field between double quotation mark of “letter and” letter.

*^14^ Japanese databases were eligible to use both English and Japanese keywords. Following keywords of "A-RU-ROH-SU" means allulose in Japanese and "PU-SHI-KOH-SU" means psicose in Japanese were added to searches at Japanese databases.

In addition to the above, we searched Matsutani Library as a pioneer of allulose commercial production company which conducted many clinical trials, and other publicities for grey literature on April 7, 2022.

### 2.4 Data collection and analysis

The study was performed using RevMan version 5.4 software for data collection and analysis.

For all studies found from databases and grey literatures, two reviewers, YT and MT, excluded duplicates.

Both reviewers went over each study independently by their titles, abstracts, and other criteria to determine inclusion for further analysis or not as the first screening. When a conflict between the above reviewers arose in the first screening, then the study was included for further analysis anyhow. Full study information was collected for all included studies after the first screening. Then, two reviewers, YT and MT, separately determined the inclusion of each study. When studies were excluded at the second screening then further rationale was indicated for those exclusions. When there was a conflict between both reviewers, then HY acted as the tie breaker for the study inclusion as the judge. A rationale of exclusion was recorded when it happened.

After the second screening, YT extracted data into a form with the name of study, the year of study, the authors’ name, the type of study design, the type of patients, the no. of patients, the allulose intake amount(s), the form of allulose, the types of meals and their quality and quantity, the duration of blood glucose monitoring, the types of blood glucose level markers, the AUC of blood glucose level markers of mean differences (MDs) with standard deviations (SDs) or standard errors (SEs), the location, and the source of funding. MT confirmed that the extraction was correct.

Two reviewers reviewed their risk of bias individually for Random sequence generation (selection bias), Allocation concealment (selection bias), Blinding of participants and personnel (performance bias), Blinding of outcome assessment (detection bias), Incomplete outcome data (attrition bias), Selective reporting (reporting bias), and Other bias. When a conflict arose between the two reviewers after each review, advisor HY became the third reviewer to be the tie breaker and make the final decision. When all above bias was assessed, results were provided in a summarized figure across studies. The result of the assessment didn’t affect the further analysis. It must present all studies and provide a narrative discussion of risk of bias. When there were studies with the “high risk” of bias, and exclusion of such study resulted in different outcome, then present them into the sensitivity analysis at the section 3.7.

Incremental AUC of blood glucose level markers for two hours were selected for measures of treatment effects.

For the preparation of a primary study question, one group consuming less than or equal to 10g (< = 10g) of allulose was defined from each study. When there were multiple experiments within one study, then they were separated into two different studies with “Study a” and “Study b” for further analysis. When there were multiple groups with different intake levels i.e. crossover trials, then we have selected one group with close to 10g allulose intake, but no more than 10g allulose intake from each study to form a “Normal-Intakes” group. When there were multiple test meals within a study, then the group highest in carbohydrates and lowest in fat or protein was chosen to avoid any influence from other nutrients. Fat is a good example as it is known to lower blood glucose levels if patients consume it with carbohydrates.

For a secondary study question, one group consuming less than or equal to 5g (< = 5g) of allulose was defined from each study. Just like “Normal-Intakes”, one group needed to be picked from each study with close to 5g allulose intake, but no more than 5g allulose intake to form a “Low-Intakes”. All conditions other than intake levels were the same as the preparation of primary study question i.e. multiple meals situation.

For both “Normal-Intakes” and “Low-Intakes” groups, all outcomes i.e. MDs, SEs, SDs, were extracted as MD with 95% confidence intervals (CIs) and standardized mean difference (SMD) with 95% CIs for further analysis.

When there was missing data from the study, the authors’ group was contacted for the missing data. If the missing data could not be recovered, then as much as possible was included in the study. If none of data could be recovered, then it was excluded with a written comment.

Heterogeneity was checked by chi square test and I^2^ statistic. Those values were considered to determine if the meta-analysis had a considerable issue or not.

The reporting biases were assessed by a funnel plot, if those numbers of studies were more than 10. When there were less than 10 studies, then a preliminary funnel plot was performed with a clear comment indication of the analysis being "Preliminary".

A Meta-analysis was performed for both “Normal-Intakes” and “Low-Intakes” in order to see the difference between the two for further considerations.

Once analysis was done, then possible subgroup analysis was indicated and conducted if necessary. When the chi square test and I^2^ statistic results had a considerable issue, then the need for further discussion and conclusion for the heterogeneity was indicated.

A Sensitivity analysis was performed to report any difference of the result caused by single study or not.

## 3. Results

### 3.1 Results of the search

**Fig 1:**
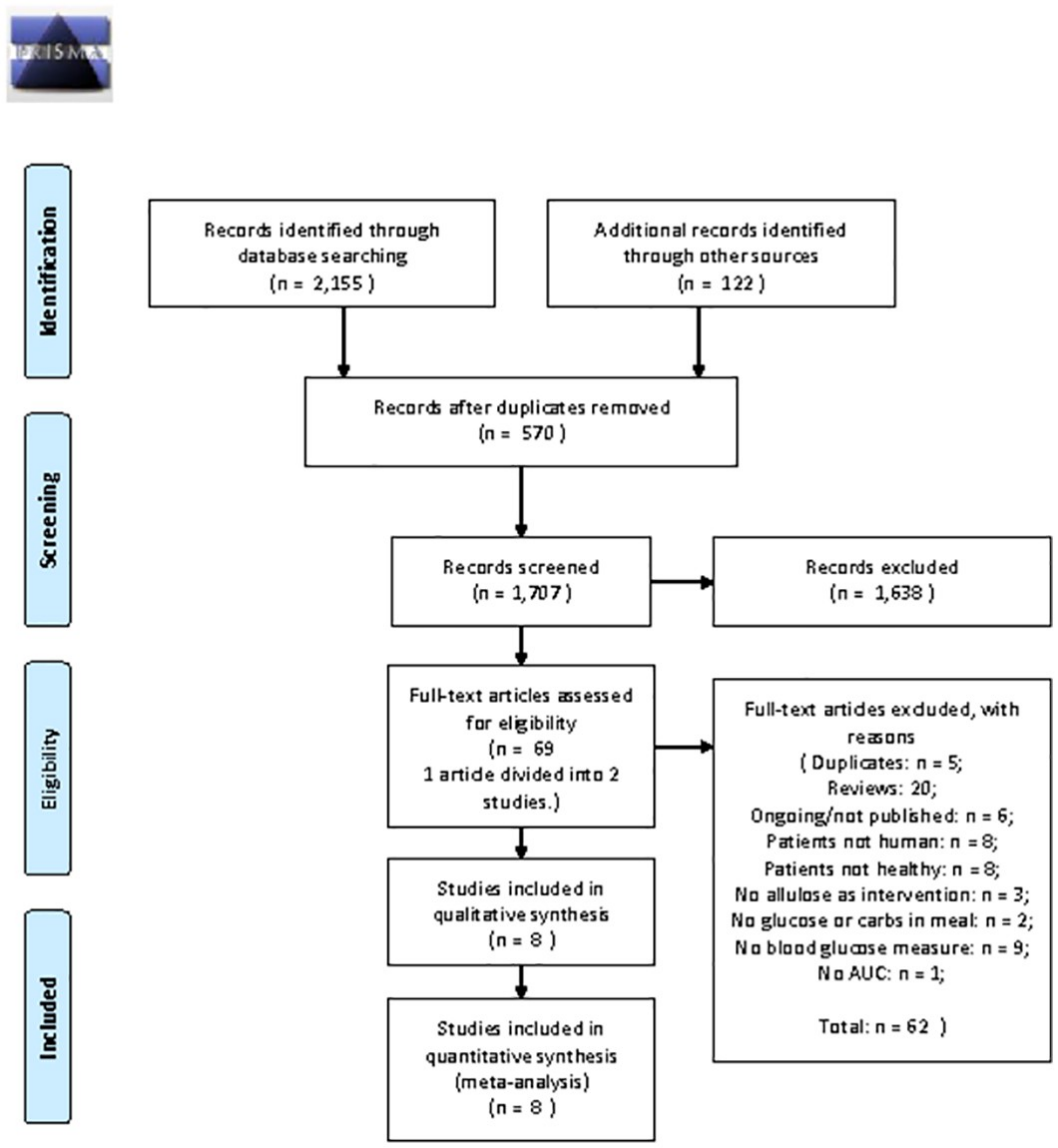
PRISMA 2009 flow diagram. *From*: Moher D, Liberati A, Tetzlaff J, Altman DG, The PRISMA Group (2009) *P*referred *R*eporting *I*tems for *S*ystematic Reviews and Meta-*A*nalyses: The PRISMA Statement. PLoS Med 6(7): e1000097. doi:10.1371/journal.pmed.1000097 **For more information, visit** www.prisma-statement.org.

A total of 2,277 articles "996 from CINII, 493 from Embase, 395 from PubMed, 233 from Ichushi Web, and 38 from CENTRAL" were obtained from the searches for all of those databases and gray literatures "122 from gray literatures includes Matsutani Library" (Fig 1). Within those 2,277 articles, 570 articles were duplicates. From the remaining 1,707 articles, 1,638 articles were excluded by review of their titles and abstracts on first screening. 69 articles were fully reviewed on second screening. During the second screening, Matsuo 2013 study [43] was subdivided into Matsuo 2013a and 2013b studies according to the protocol as two experiments were in the study. 62 articles were excluded by the second screening, and the remaining 8 studies from 7 articles were included. Their data was extracted for further analysis [13, 27, 43, 49–52].

### 3.2 Included studies

**Fig 2:**
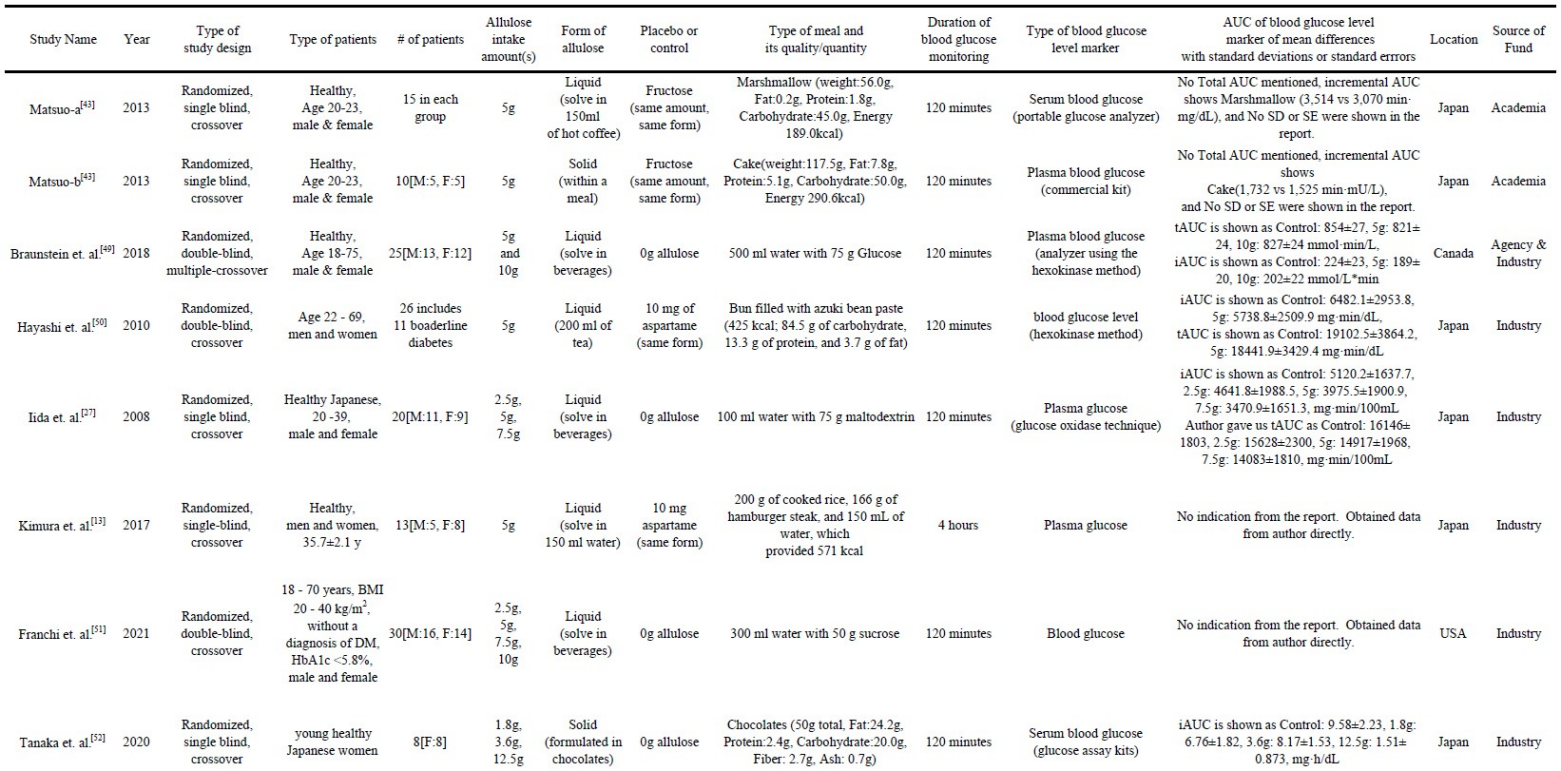
Data extracted from included studies.

Fig 2 shows descriptions of the data extracted from the included studies. Both Matsuo 2013a and 2013b experiments were missing SD and SE. Based on the protocol, the largest possible SD was calculated mathematically for further analysis. 8 studies and their data were extracted based on the protocol.

A total of 145 patients per group participated with a range of patients from 8–30 per group. All experiments were small, but randomized, blinded "single or double", and crossover. 6 out of 8 experiments used allulose in the form of a liquid and two experiments used allulose in the form of a solid. 7 out of 8 experiments had a group of 5g of allulose intake. Two experiments used the same amount of fructose, two experiments used 10 mg of aspartame, and four experiments used no allulose or no intake as a comparator.

### 3.3 Excluded studies

62 articles were excluded during the second screening. Five studies were duplicates. Six studies were on-going and their full reports were not available, or had not published, or an author had retired. 20 articles were review articles. Eight studies did not meet the inclusion criteria of "the study patients are humans". Eight studies did not meet the inclusion criteria of "the study patients are healthy". Three studies didn’t use allulose as an intervention. Two studies did not meet the inclusion criteria of "the study patients consume some types of meal to increase blood glucose with the intervention". Nine studies did not meet the inclusion criteria of "the study takes blood glucose measurements for at least 2 hours". One study did not meet the inclusion criteria of "the study reports AUC of blood glucose levels or it could be obtained from the authors’ group".

### 3.4 Risk of bias in included studies

**Fig 3:**
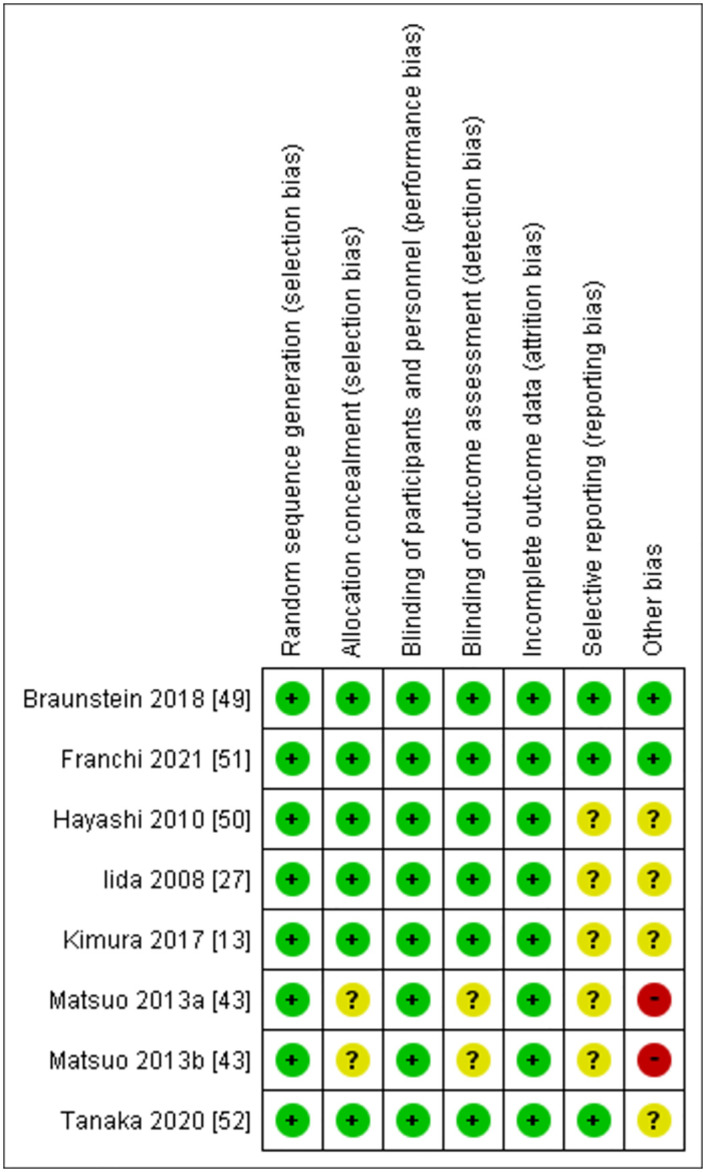
Risk of bias summary for each included study.

**Fig 4:**
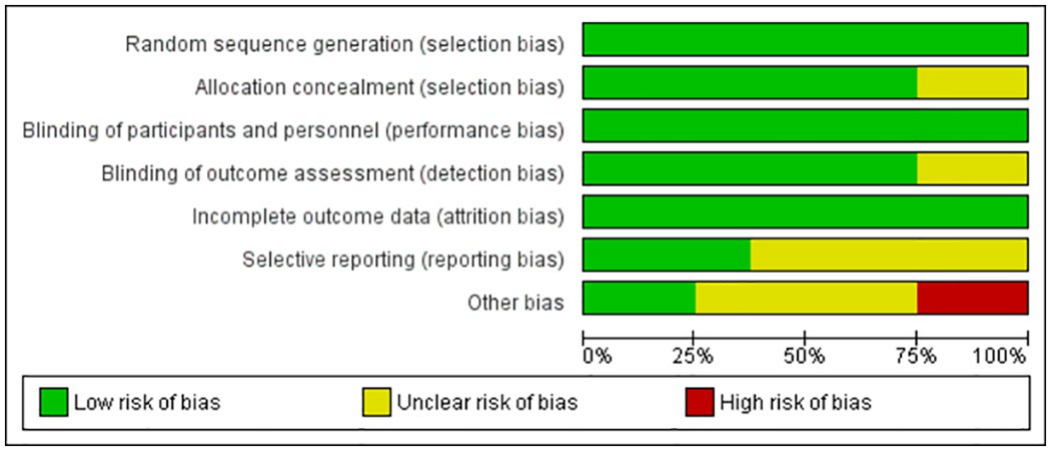
Risk of bias graph for each included study.

The risk of bias in the included studies were assessed as Figs 3 and 4.

All studies were "Randomized" and single or double "Blinded". With that being mentioned in each study, some studies didn’t report their method of "Randomized" or "Blinded". As the results, some "selection bias" and “detection bias” were assessed as "Unclear Risk". All studies seem to have reported all data which gives a low risk of incomplete outcome data as "attrition bias". Some studies didn’t register prior to their start, and there were some risks at the reporting bias.

**Fig 5:**
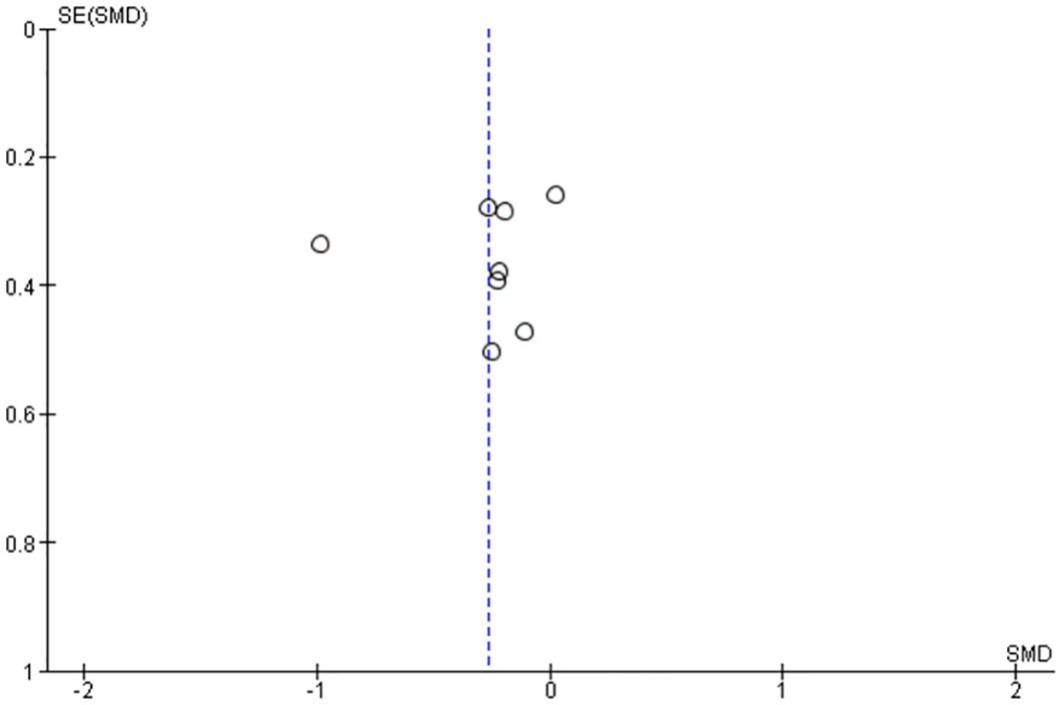
Preliminary funnel plot of incremental AUC at the selected studies for Normal-Intakes "<10g" and control.

**Fig 6:**
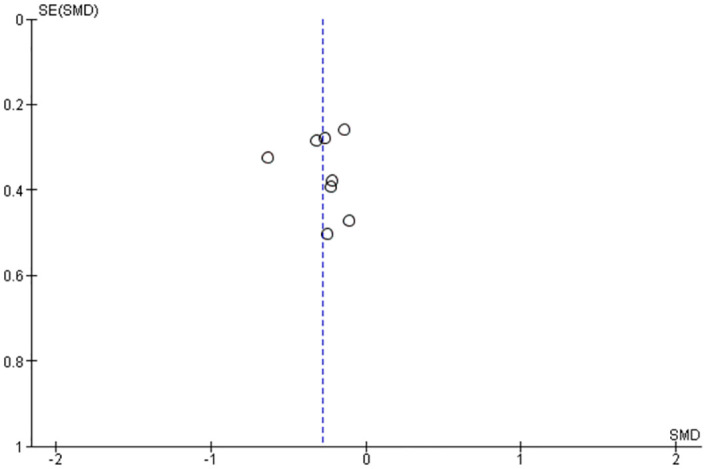
Preliminary funnel plot of incremental AUC at the selected studies for "<5g" Low-Intakes and control.

In addition to above, the reporting biases were separately assessed by a funnel plot for each preliminary information as Figs 5 and 6.

All funnel plots were preliminary, because the number of selected studies was less than 10.

Other bias was assessed from single-centre versus multi-centre study, study size compared to other study of nutrition, and the influence from funders. Some studies resulted in "High Risk", as the number of patients were low and missing information for some of methods, and "Unclear Risk" as some studies were missing to provide the judgement of either “High Risk” or “Low Risk”.

### 3.5 Effects of interventions

**Fig 7:**
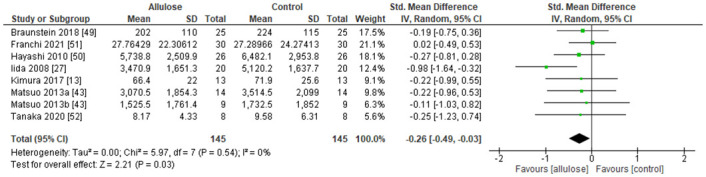
Forest plot of Random Effect Model for incremental AUC with a comparison between "Normal-Intakes (< = 10g) and control".

**Fig 8:**
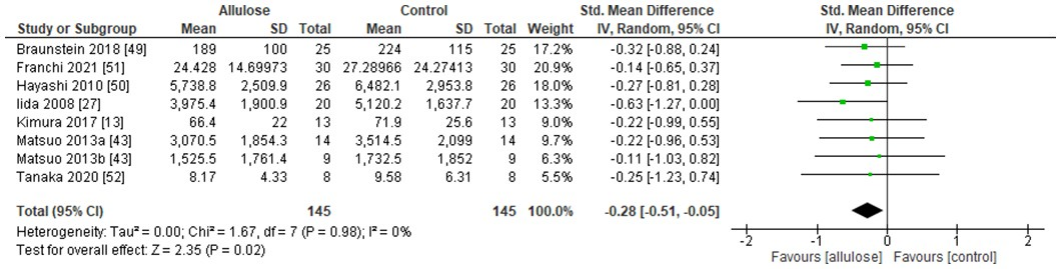
Forest plot of Random Effect Model for incremental AUC with a comparison between "Normal-Intakes (< = 5g) and control".

The meta-analysis was performed using SMD since corrected studies were used different markers for the blood glucose concentrations i.e. plasma glucose and blood glucose. Fig 7 shows the forest plot of Random Effect Model for incremental AUC with comparisons between Normal-Intakes and the Control. This model shows that the 10g intake of allulose attenuated postprandial blood glucose levels. It is significant as P = 0.03. The effect size for the model is -0.26. Fig 8 shows the forest plot of Random Effect Model for incremental AUC with comparisons between Low-Intakes and the Control. This model shows that the 5g intake of allulose attenuated postprandial blood glucose levels. It is significant as P = 0.02. The effect size for the model is -0.28.

### 3.6 Heterogeneity

For both forest plots of Figs 7 and 8, the chi square test and the I^2^ statistic were checked. The lowest number of P value from chi square tests was P = 0.54. The results of the I^2^ statistic were 0% for both. They indicate that there is no considerable issue with the heterogeneity, and no further information was considered on the heterogeneity.

### 3.7 Sensitivity analysis

From the forest plots in Figs 7 and 8, we took out each study and reanalyzed for changes in the results. Only the Iida 2008 study [27] affected the result significantly of “allulose favours” as it crossed the center line between “allulose favours” and “control favours” at both Figs. It is a small study as the number of patients was 20 per group and study of similar size may affect the results significantly.

### 3.8 Summary of findings

The Summary of findings are shown as Tables 1 and 2.

**Table 1:** Summary of findings for 10g of allulose intervention.

**10g of allulose compared to control for Postprandial Attenuation of Blood Glucose Levels in Healthy Human**					
**Patient or population**: Postprandial Attenuation of Blood Glucose Levels in Healthy Human					
**Setting**:					
**Intervention**: 10g allulose					
**Comparison**: control					
**Outcomes**	**№ of participants (studies) Follow up**	**Certainty of the evidence (GRADE)**	**Relative effect (95% CI)**	Anticipated absolute effects* **(95% CI)**	
				**Risk with control**	**Risk difference with 10g allulose**
Incremental AUC of Blood Glucose Levels	290 (8 RCTs)	⊕⊕⊕⊝ MODERATE^a^	-	The mean incremental AUC of Blood Glucose Levels was 0	SMD 0.26 lower (0.49 lower to 0.03 lower)

*The risk in the intervention group (and its 95% confidence interval) is based on the assumed risk in the comparison group and the **relative effect** of the intervention (and its 95% CI).

**CI**: Confidence interval; **RR**: Risk ratio; **OR**: Odds ratio;

**GRADE Working Group grades of evidence**

**High certainty**: We are very confident that the true effect lies close to that of the estimate of the effect

**Moderate certainty**: We are moderately confident in the effect estimate: The true effect is likely to be close to the estimate of the effect, but there is a possibility that it is substantially different

**Low certainty**: Our confidence in the effect estimate is limited: The true effect may be substantially different from the estimate of the effect

**Very low certainty**: We have very little confidence in the effect estimate: The true effect is likely to be substantially different from the estimate of effect

**Explanations**

All selected studies are small in terms of study size as they are only 8–30 patients in one group.

**Table 2:** Summary of findings for 5g of allulose intervention.

**5g of allulose compared to control for Postprandial Attenuation of Blood Glucose Levels in Healthy Human**					
**Patient or population**: Postprandial Attenuation of Blood Glucose Levels in Healthy Human					
**Setting**:					
**Intervention**: 5g allulose					
**Comparison**: control					
**Outcomes**	**№ of participants (studies) Follow up**	**Certainty of the evidence (GRADE)**	**Relative effect (95% CI)**	Anticipated absolute effects* **(95% CI)**	
				**Risk with control**	**Risk difference with 5g allulose**
Incremental AUC of Blood Glucose Levels	290 (8 RCTs)	⊕⊕⊕⊝ MODERATE^a^	-	The mean incremental AUC of Blood Glucose Levels was 0	SMD 0.28 lower (0.51 lower to 0.05 lower)

*The risk in the intervention group (and its 95% confidence interval) is based on the assumed risk in the comparison group and the **relative effect** of the intervention (and its 95% CI).

**CI**: Confidence interval; **RR**: Risk ratio; **OR**: Odds ratio;

**GRADE Working Group grades of evidence**

**High certainty**: we are very confident that the true effect lies close to that of the estimate of the effect

**Moderate certainty**: we are moderately confident in the effect estimate: the true effect is likely to be close to the estimate of the effect, but there is a possibility that it is substantially different

**Low certainty**: our confidence in the effect estimate is limited: the true effect may be substantially different from the estimate of the effect

**Very low certainty**: we have very little confidence in the effect estimate: the true effect is likely to be substantially different from the estimate of effect

**Explanations**

All selected studies are small in terms of study size as they are only 8–30 patients in one group.

## 4. Discussion

This study is conducted in order to see the effect of allulose to postprandial blood glucose levels in healthy humans by systematic review and meta-analysis. In healthy adults, 10g or 5 g of allulose, added to a carbohydrate-containing meal showed to lower postprandial incremental AUC for glucose, compared with the same meal without allulose, over the postprandial period in an intervention trial setting.

From all allulose related articles searched throughout different databases, 8 experiments from 7 different articles were selected for meta-analysis. The total number of experiments included in this study was small compare to other systematic reviews. The level of significance could be influenced by any new study. According to previous research, followings are estimated mechanisms, which leads to an attenuation of glycemic response. Matsuo and Izumori found that allulose inhibits α-glucosidase activities which leads to suppression of glycemic responses after carbohydrate ingestion [21]. In the small intestine, allulose and other monosaccharides such as glucose and fructose share the same transporters such as GLUT2 and GLUT5. One study indicates slower absorption of glucose if allulose is also present and vice versa [28]. Glucose and allulose are likely sharing a transporter as they are same in terms of molecular formula, C_6_H_12_O_6_. Allulose stimulates glycogen synthesis in the liver [29], which causes faster restoration of glycogen in the liver and muscle after exercise [30]. Since the glycogen is formed from glucose, this effect also reduces the availability of glucose in blood. Allulose promotes this effect. Allulose also induces Glucagon-like peptide-1 (GLP-1) release from intestinal L-cells, and regulates glucose concentrations after glucose and allulose intake [31, 32]. With the multiple reasons above, allulose attenuates blood glucose increases from glucose source intake without causing hypoglycemia. Its effect size is SMD -0.26 to -0.28 from different intakes. It is clinically significant and small by using the suggestion of Cohen as 0.2 is a small effect, 0.5 is a moderate effect, and 0.8 and above are large effects. When we convert back to the Braunstein 2018 [49] study which has the lowest risk of bias from all included studies. Then, there is about 13–14% reduction of incremental AUC from the Control. Its effect size is lower than a major blood glucose lowering drug like Metformin [53, 54]. The mechanisms of the effect seem different. The small effect is better in terms of its safety since allulose is just a naturally occurring food ingredient.

The risk of bias was assessed, there are some high and unclear risks of bias to the overall studies. The forest plots were checked for heterogeneity with the chi square test and I^2^ statistics. Both numbers show there is no issue with the heterogeneity.

The funnel plots were performed to check a part of the reporting bias and a part of the heterogeneity, and there were low risk of the reporting bias and no issues with heterogeneity. These are preliminary results as their number of experiments are less than 10 for each plot, but good enough to indicate that there is no issue at this point.

The study question was set to find for two different outcomes. Which were intervention dose levels comparing 5g and 10g. There are only two experiments of 7.5g dose group and 10g dose group which we can find for more than 5g dose group. They make very similar outcomes between primary study question and secondary study question as 5 out of 8 inputs are the same.

The way of extracting an experiment from a study can be different such as with Matsuo 2013a study [43]. In Matsuo 2013a, different meal types were used, and the weight of this study could triple if they are all included [43]. The level of significance may be higher if that data is obtained.

The sensitivity analysis shows that one study by Iida et al. [27] can make a significant result. It means that the strength of this study result is not concrete. One study with similar size may affect the whole result and conclusion.

The previous study by Braunstein et al. [42] reports a systematic review and meta-analysis of 6 experiments from 5 studies. Their study uses a different formula to search and select experiments compared to this study i.e. this study searches Japanese database written in Japanese language. Their study includes an experiment with diabetic patients. This study excluded patients with diabetes to see an affect only on healthy humans. Their study includes all dosage groups less than or equal to 10g, and we select one group close to less than 10g from each experiment. This study has an additional group consuming less than 5g in order to see dose effect.

There are some similarities between the two studies. Both studies share same result from their meta-analysis that allulose contributes to less incremental AUC compared to their controls.

From the comparison of incremental AUC for the “Normal-Intakes” group and the “Low-Intakes” group, this effect may not associate with dose dependent. Low-Intake group has larger effect size of -0.28. This result mainly comes from a study which experienced by Franchi et al. [51] as it shows that there is no significant effect at 10g intake. Other studies show that there is more effect if the patients take 10g of allulose. Further studies are needed to confirm that the effect is dose dependent or not.

There is one experiment which contradicts the above result as Matsuo 2013b [43] has a larger number of AUC compared to its control. This may have happened because of a dispersion of allulose within a body. Livesey et al. [55] mentioned that the difference between a solid and a liquid caused the postprandial blood glucose attenuation effect to be different. This study uses resistant maltodextrin. With our selected experiments, Matsuo 2013b [43] used allulose in a solid form when all other experiments used allulose in a liquid form. This means that a dispersion of test materials may not be great enough after their intake. A low dispersion of allulose may reduce the effect.

There are limitations to the study since the number and size of studies are small. Any future study with larger size may be needed to confirm the study findings. Another limitation is that the protocol doesn’t look for other blood glucose related markers like insulin and GLP-1. We may have an additional outcome, if we look after those parameters in the future.

Even though there are limitations to the study, allulose may be a key ingredient for the Sugar Substitution. Since allulose by itself is a Sugar and has its good property of sweet taste, the use of allulose may be called “Sugar Reformulation” and best approach for the Sugar Reduction.

## 5. Conclusion

### 5.1 Implications for practice

From the study result, allulose intake attenuates postprandial blood glucose levels in healthy humans. A 5g intake of allulose per meal is significant enough to attenuate postprandial blood glucose levels in healthy humans. A 10g intake of allulose attenuate postprandial blood glucose levels in healthy humans as well.

### 5.2 Implications for research

The sensitivity analysis shows that one study could be significant enough to change the outcome. When an update occurs, a method of the study could be modified with more efficient searches, different ways of inclusion and exclusion, and finding different outcomes. Dose difference of allulose may result in a different effect size for the attenuation effect of postprandial blood glucose levels in healthy humans. The effect may differ by race as well as gender. Also, there might be a difference between a liquid form and a solid form. Additional considerations that factor into effects are the timing of an intake and its ratio of allulose in a meal. Insulin and GLP-1 could be another considerable marker for the meta-analysis, but number of studies are less than the blood glucose.

## Supporting information

S1 File PRISMA 2009 checklist page 1. (TIF)

S2 File PRISMA 2009 checklist page 2. (TIF)

S1 Checklist PRISMA 2009 checklist. (DOC)

## Abbreviations

allulose: D-allulose
AUC: Area Under Curve
CENTRAL: Cochrane Central Register of Controlled Trials
CINII: Citation Information by National Institute of Informatics
EMBASE: Excerpta Medica Database
GLP-1: Glucagon-like peptide-1
GLUT: glucose transporter
ICHUSHI: Japan Medical Abstracts Society
MD: mean difference
MEDLINE: US National Library of Medical Database
NCDs: noncommunicable diseases
RevMan: Cochrane Handbook and Review Manager
SD: standard deviation
SE: standard error
SMD: standardized mean difference
WHO: World Health Organization
