# Supplementary figures and images for: Allulose for the attenuation of postprandial blood glucose levels in healthy humans: A systematic review and meta-analysis
Source: PLoS One. 2023 Apr 6;18(4):e0281150. doi: 10.1371/journal.pone.0281150 (PMC10079081; doi:10.1371/journal.pone.0281150)

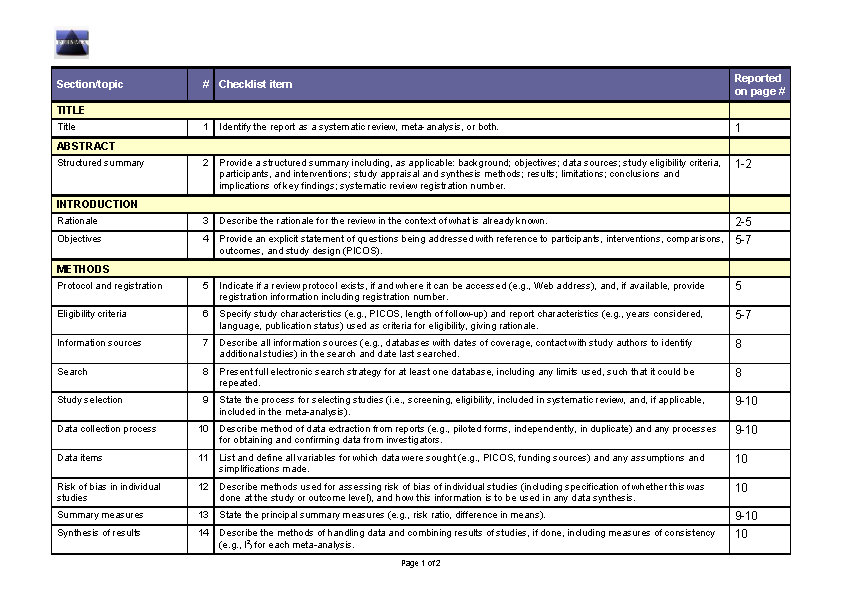

Supplement: S1 File — (TIF) [file pone.0281150.s001.tif]

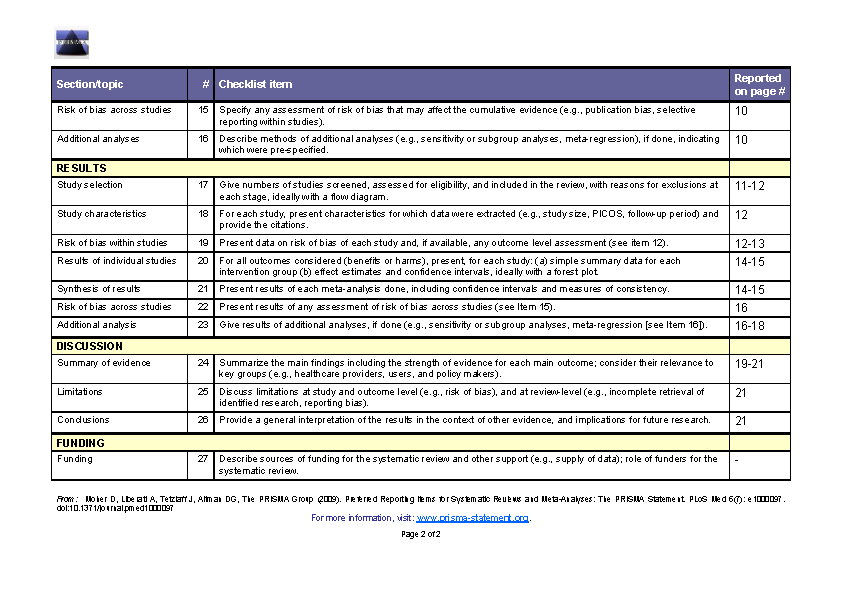

Supplement: S2 File — (TIF) [file pone.0281150.s002.tif]
